# Deep spectral learning for label-free optical imaging oximetry with uncertainty quantification

**DOI:** 10.1038/s41377-019-0216-0

**Published:** 2019-11-20

**Authors:** Rongrong Liu, Shiyi Cheng, Lei Tian, Ji Yi

**Affiliations:** 1 Department of Biomedical Engineering, Northwestern University, Evanston, IL 60208 USA; 2 Department of Electrical and Computer Engineering, Boston University, Boston, MA 02215 USA; 3 Department of Biomedical Engineering, Boston University, Boston, MA 02215 USA; 4 Department of Medicine, Boston University School of Medicine, Boston Medical Center, Boston, MA 02118 USA

**Keywords:** Imaging and sensing, Optical spectroscopy, Interference microscopy

## Abstract

Measurement of blood oxygen saturation (*s*O_2_) by optical imaging oximetry provides invaluable insight into local tissue functions and metabolism. Despite different embodiments and modalities, all label-free optical-imaging oximetry techniques utilize the same principle of *s*O_2_-dependent spectral contrast from haemoglobin. Traditional approaches for quantifying *s*O_2_ often rely on analytical models that are fitted by the spectral measurements. These approaches in practice suffer from uncertainties due to biological variability, tissue geometry, light scattering, systemic spectral bias, and variations in the experimental conditions. Here, we propose a new data-driven approach, termed deep spectral learning (DSL), to achieve oximetry that is highly robust to experimental variations and, more importantly, able to provide uncertainty quantification for each *s*O_2_ prediction. To demonstrate the robustness and generalizability of DSL, we analyse data from two visible light optical coherence tomography (vis-OCT) setups across two separate in vivo experiments on rat retinas. Predictions made by DSL are highly adaptive to experimental variabilities as well as the depth-dependent backscattering spectra. Two neural-network-based models are tested and compared with the traditional least-squares fitting (LSF) method. The DSL-predicted *s*O_2_ shows significantly lower mean-square errors than those of the LSF. For the first time, we have demonstrated *en face* maps of retinal oximetry along with a pixel-wise confidence assessment. Our DSL overcomes several limitations of traditional approaches and provides a more flexible, robust, and reliable deep learning approach for in vivo non-invasive label-free optical oximetry.

## Bioimaging: Artificial intelligence casts an eye on microvascular maps

Optical imaging of blood oxygen saturation (*s*O_2_) within the retinal circulation can be performed with higher confidence using machine learning techniques. Advanced imaging methods that detect the spectral contrast of haemoglobin have enabled researchers to measure *s*O_2_ levels in the microvascular system without invasive procedures. A team from Boston University lead by Drs. Ji Yi and Lei Tian have now developed a deep learning framework that estimates simultaneously *s*O_2_ level and uncertainties due to experimental variabilities, such as tissue scattering. The team compiled data from recent literature to train a neural network that quantify *s*O_2_ levels from spectral features in the blood vessels of eyes. Subsequent testing demonstrated this approach can remove the need for analytical models for spectral fitting, and map out regions where uncertainty of the predictions are high.

## Introduction

Microvascular systems deliver oxygen to support cellular metabolism and maintain biological functions. Within the local microenvironment of blood vessels, oxygen unloads from haemoglobin and diffuses freely from red blood cells (RBCs) to tissues following the gradient of oxygen partial pressure (*p*O_2_), which determines the oxygen saturation (*s*O_2_) of haemoglobin. The measurement of microvascular *s*O_2_ can thus help in assessing the local tissue oxygenation and provide invaluable insight into local tissue metabolism, inflammation, and oxygen-related pathologies. It can also offer diagnosis and prognosis for several major diseases, such as cancers, diabetic milieu and complications, cardiovascular diseases, dementia, etc ^1–5^.

**Fig. 1:**
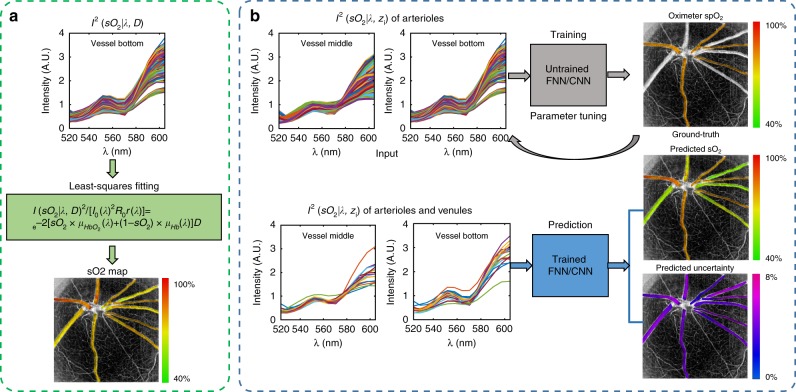
Methods for calculating retinal blood vessel *s*O_2_ by **a** the traditional LSF and **b** our neural-network-based DSL with uncertainty quantification. Traditional LSF optimizes a rigid parametric model to best fit the spectral measurements for sO_2_. DSL bypasses any rigid models and trains neural networks with paired arterial spectra and oximeter *sp*O_2_ readings as the ground truth. After training, the neural network models predict both *s*O_2_ and its uncertainty.

In recent years, several non-invasive and label-free optical-imaging oximetry techniques have been developed to measure microvascular *s*O_2_. Despite their differences, the fundamental mechanism is the same, being based on the *s*O_2_-dependent spectral contrast from haemoglobin^6^. The spectral measurement is then related to *s*O_2_ through a complex physical model incorporating tissue geometry, heterogeneous tissue scattering, light attenuation and propagation, and imaging optical instruments. This model is often simplified and analytically formulated under different approximations and assumptions. Examples include spatial frequency domain imaging^7,8^ in the diffusive regime under the P3 approximation, multi-wavelength imaging^9–12^ and visible light optical coherence tomography (vis-OCT)^13–15^ in the ballistic regime based on Beer’s law combined with the first Born approximation^16–25^, photoacoustic microscopy/tomography assuming a uniform laser fluence inside the tissue^26,27^, and photothermal imaging assuming a linear relation between the blood absorption and the change in the optical signal^28–31^. The *s*O_2_ estimation thus requires solving an ill-posed inverse problem that is inevitably subject to model inaccuracies, noise, systemic spectral bias, and experimental conditions. One widely used inversion method is the spectral least-squares fitting (LSF), which estimates the *s*O_2_ by matching the spectral data with the analytical model, as shown in Fig. 1a. However, multiple sources of spectral errors exist in practice that are impossible to fully parameterize in an analytical form, which in turn compromises the *s*O_2_ estimation accuracy, repeatability, and cross-comparison between different devices, test subjects, and time. Therefore, it is imperative to develop a more robust model to enable more accurate quantification of microvascular *s*O_2_ for label-free optical-imaging oximetry.

In this work, we develop a new data-driven deep spectral learning (DSL) method to enable highly robust and reliable *s*O_2_ estimation, as shown in Fig. 1b. By training a neural network to directly relate the spectral measurements to the corresponding independent *s*O_2_ labels, DSL bypasses the need for a rigid parametric model, similar to existing deep-learning methods for solving optical inverse problems^32–36^. We show that DSL can be trained to be highly robust to multiple sources of variabilities in the experiments, including different setups, imaging protocols, speeds, and other possible longitudinal variations.

An essential feature of our DSL method is uncertainty quantification. Due to biological variations and tissue heterogeneity, an assessment of the reliability of each *s*O_2_ measurement is crucial in clinical applications and for guarding against vulnerabilities in making *overly confident* predictions when imaging rare cases^37^. Existing model-based methods generate a single value of *s*O_2_ for each spectral measurement, i.e., a point estimate. The accuracy and uncertainty of the point estimate can be assessed only by taking repeated measurements against the ground truth in a well-controlled experiment. This uncertainty estimation presents a clear limitation for many biomedical applications in which the ground truth is often inaccessible in vivo, and the statistical analysis can be performed only retrospectively on those repeated measurements^13,15,18,31,38^. Instead of assessing the variabilities in the data retrospectively, we develop our DSL model based on an uncertainty learning framework^36^ to encapsulate the statistics in the learned model, essentially shifting the burden of repeated measurements in the model-based methods to the training phase of DSL. After the training, the DSL model predicts both *s*O_2_ and its tandem standard deviation, assessing the uncertainty for each *s*O_2_ prediction (i.e., a statistical distribution describing all possible *s*O_2_ levels of each prediction given the measurements). Most importantly, we show that the DSL-predicted statistics closely match those obtained from ensemble calculations. This means that the confidence level calculated from the DSL prediction can be used as a surrogate estimate to the true accuracy of the estimate, making DSL reliable.

**Fig. 2:**
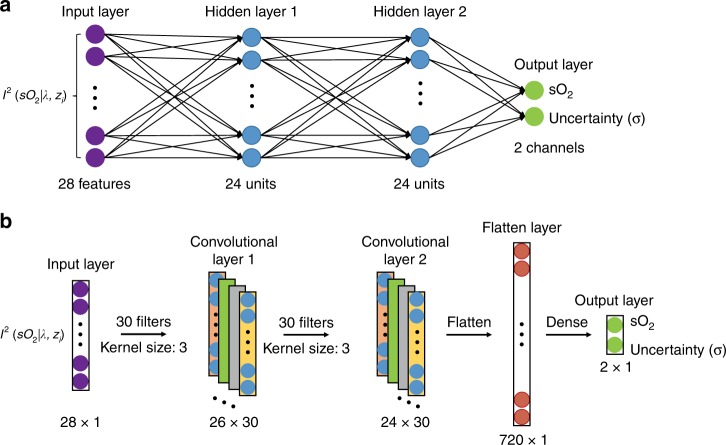
Structures of the FNN model **a** and the CNN model **b** for *s*O_2_ prediction, with uncertainty quantified by the predicted standard deviation *σ*.

We demonstrate DSL using two sets of vis-OCT experiments for oximetry on rat retinas from refs. ^13,15^. Vis-OCT in rodent and human retinas has been extensively demonstrated in recent years^19,39–42^, and several studies have shown significant clinical potential in the diagnosis and prognosis of several major retinal diseases using oximetry^43–47^. Two DSL models are investigated, including a 1D fully connected neural network (FNN) and a 1D convolutional neural network (CNN), the network architectures of which are shown in Fig. 2a, b, respectively. Our results show that both DSL models significantly outperform the LSF, in terms of both the estimation accuracy and the robustness to experimental variations. We further conduct a quantitative statistical analysis based on uncertainty learning to establish the confidence level of the two proposed models and further justify the reliability of DSL. Finally, imaging oximetry is demonstrated on *en face* *s*O_2_ maps of rat retinas along with the corresponding uncertainty maps, providing a visualization of the DSL predictions. This process allows us to assess the accuracy of the prediction based on the underlying physiological conditions.

## Results

### Data source

**Fig. 3:**
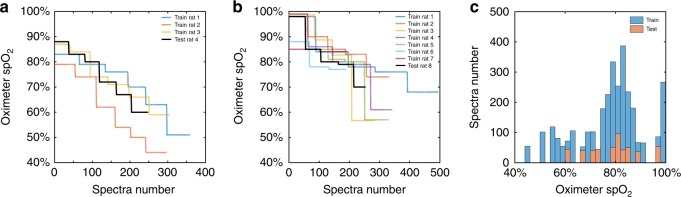
Oximeter *sp*O_2_ readings (ground truth labels) for training and testing. **a** The readings of rat retinal arterioles from normoxia to hypoxia from ref. ^13^. **b** The readings of rat retinal arterioles from hyperoxia, 5% CO_2_, normoxia to hypoxia from ref. ^15^. **c** Histograms of all *sp*O_2_ readings for both the training and testing sets.

To evaluate the effectiveness of the DSL approach, we compiled two datasets from the previous literature on vis-OCT retinal oximetry^13,15^. Specifically, the data in Fig. 3a are from ref. ^13^, and that in Fig. 3b, from ref. ^15^. Both datasets used similar experimental protocols in which the oxygen content in the ventilation gas was adjusted to induce systemic hypoxia or hyperoxia, and vis-OCT measurements were taken under each ventilation condition. In ref. ^13^, four rats were measured under six ventilation conditions, from normoxia (21% O_2_, 79% N_2_), to five hypoxia challenges, in which the oxygen content was reduced step-wise: step 1 (19% O_2_, 81% N_2_), step 2 (17% O_2_, 83% N_2_), step 3 (15% O_2_, 85% N_2_), step 4 (13% O_2_, 87% N_2_), and step 5 (9% O_2_, 91% N_2_). In ref. ^15^, eight rats were measured under five ventilation conditions, sequencing from normoxia, hyperoxia (100% O_2_), 5% carbon dioxide (21% O_2_, 74% N_2_, 5% CO_2_), hypoxia (10% O_2_, 90% N_2_), and finally to normoxia. Under each ventilation condition, the systemic arterial *sp*O_2_ reading was taken by a pulse oximeter attached to a rear leg of each rat. All the vis-OCT and pulse oximetry measurements were taken approximately one minute after the ventilation transition, when the *sp*O_2_ readings were stable. The *sp*O_2_ readings are used as the ground truth label for the major retinal arterioles for neural network training. Depth-dependent backscattering spectra of rat retinal arterioles in vis-OCT were extracted under each ventilation condition as spectral input to the neural network. The extracted arteriole spectra with the spO_2_ labels were then split into training and testing sets. In ref. ^13^, data from three rats were used as the training sets, with the remaining one as the testing set. In ref. ^15^, data from seven rats were used as the training sets, and the remaining one was the testing set. We mixed the training data from both studies to train the DSL models and make predictions on the testing data from both studies. All the training/testing and the subsequent data analysis are identical for CNN and FNN to compare our DSL approach with the standard LSF. Figure 3a, b summarize the number of spectra extracted and the corresponding *sp*O_2_ labels in descending order, respectively. Figure 3c shows the histogram of all the training and testing spectra with their corresponding *sp*O_2_ labels.

### Prediction of arterial sO_2_

**Fig. 4:**
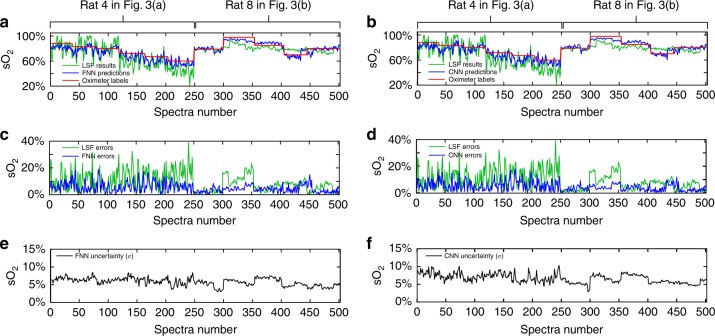
Rat retinal arteriolar sO_2_ of the testing data under different ventilation conditions predicted by the FNN, CNN, and LSF models. *s*O_2_ predicted by the FNN **a** and CNN **b**, compared with the oximeter *sp*O_2_ readings and LSF calculations. Errors of *s*O_2_ predicted by the FNN **c** and the CNN **d** model compared to the LSF results. Predicted uncertainties of *s*O_2_ obtained by the FNN **e** and the CNN **f** model measured by the standard deviations (*σ*). The first 248 spectra were from ref. ^13^, and the rest were from ref. ^15^.

Once the networks are trained, the *s*O_2_ predictions from the testing set obtained by a FNN and CNN are plotted in Fig. 4a, b, respectively, along with the ground truth oximeter *sp*O_2_ readings and the LSF estimates for comparison. Our DSL models were trained by a combination of both datasets from refs. ^13^,^15^. We optimized the LSF model parameters on the same data pool to ensure a fair comparison between the two methods. The detailed descriptions of the LSF model and parameter optimization are provided in the “Materials and methods” section. The first 248 testing spectra are from ref. ^13^, and the remaining 254 are from ref. ^15^. In general, the *s*O_2_ estimations made using both DSL models (FNN and CNN) and LSF agree with the *sp*O_2_ readings. A closer look reveals that DSL predictions have lower variations than that of LSF, which we attribute to the DSL’s improved robustness to noise and other random signal fluctuations. The absolute errors between the *s*O_2_ predictions and the corresponding *sp*O_2_ are plotted in Fig. 4c, d, respectively. Errors from both DSL models are significantly lower than that of the LSF model. To quantitatively compare the three different models, we calculate the mean square errors (MSEs) of the FNN and CNN models to be 0.3539 × 10^−2^ and 0.3200 × 10^−2^, respectively, both of which are <1/3 the MSE from LSF (1.358 × 10^−2^). An important feature of our DSL models is its ability to quantify uncertainty via the tandem standard deviation (*σ*) for each *s*O_2_ prediction (Fig. 4e, f). Overall, both FNN and CNN predict *σ* to be ~5–7%, and the variation in *σ* justifies the use of a *heterogeneous* model in our customized loss function. We also see that the prediction for ref. ^15^ has slightly lower variation in *σ* than that in ref. ^13^, presumably due to higher animal numbers and larger training datasets (Fig. 3). Out of the 2779 training spectra, 1930 are from ref. ^15^.

To demonstrate the generality of our DSL approach, we trained DSL and optimized LSF for each of the two datasets separately and repeated the above comparisons. The accuracies of the two DSL models are consistently better than that of LSF (Figs. S1 and S2) by approximately three times (Table S1). Even when we used data from one reference for training/optimization and tested models on the data from the other reference, DSL still outperformed LSF by more than four times (Fig. S3, Table S1). Interestingly, the uncertainty estimated by DSL increases significantly to ~10% when the networks are tested on a completely new data source (Fig. S3), truthfully reflecting the lower confidence levels of the two networks in this situation.

### Evaluation of the quantified uncertainty

Our uncertainty quantification assumes that the predicted sO_2_ follows a *heterogeneous* Gaussian distribution given different spectral inputs. To validate our uncertainty metrics, we retrospectively calculated the actual probability that the ground truth (*sp*O_2_) falls within a certain confidence interval of the predicted sO_2_ and summarize the results using the reliability diagram^33,36,48^. To construct a reliability diagram, we gather a sub-set of predictions with a specific standard deviation *σ*_0_. We then calculate the probability from this sub-set of data that satisfy the criterion of |[*s*O_2_]_*i*_ – [*sp*O_2_]_*i*_| < *ησ*_0_, where [*s*O_2_]_*i*_ and [*sp*O_2_]_*i*_ are the prediction and the corresponding ground truth from the *i*th vis-OCT spectrum, respectively:

1 $${P}\left( {\sigma _0,\eta } \right) = \frac{1}{{\left| {S_{\sigma _0}} \right|}}\mathop {\sum}\limits_{i \in S_{\sigma _0}} {{\rm{I}}_{\left\{ {\left| {\left[ {s{\mathop{\rm{O}}\nolimits} _2} \right]_i - \left[ {sp{\mathop {\rm{O}}\nolimits} _2} \right]_i} \right| < \eta \sigma _0} \right\}}}$$

where *η* is a variable that defines the confidence interval and *S* denotes the sub-set of the prediction with the specified standard deviation. In practice, we relaxed *σ*_0_ to *σ*_0_ ±1% to include sufficient data to ensure reliable statistical calculations. Intuitively, the probability will approach 1 when *η* increases, i.e., a larger error tolerance. At the same time, *η* has a one-to-one correspondence to the theoretical confidence value calculated from the normal distribution. The reliability diagram essentially plots the actual probability against *η* or the theoretical confidence. For an ideal well-calibrated model, the actual probability *P*(*σ*_0_, *η*) should equal the theoretical confidence—falling on the diagonal line in the graph. When the actual probability *P*(*σ*_0_, *η*) is lower than the theoretical confidence, it indicates that the model is *over-confident*—the reliability curve is *under* the diagonal line. When the actual probability *P*(*σ*_0_, *η*) is higher than the theoretical confidence, it indicates that the model is *conservative*—the reliability curve is *above* the diagonal line.

**Fig. 5:**
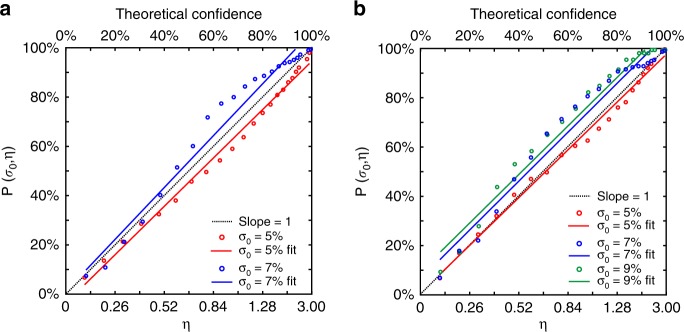
Statistical analysis of the *s*O_2_ predictions with the quantified uncertainty. **a** The linear fits of *P*(*σ*_0_, *η*) to *η* and the confidence for the FNN model when the uncertainty measured by the standard deviation (*σ*_0_) is 5% and 7%. **b** The same graph for the CNN model when the standard deviation (*σ*_0_) is 5%, 7%, and 9%.

The reliability diagrams for both models are shown in Fig. 5. To cover over 90% of the total 502 predictions of the testing data in the reliability diagram, we set *σ*_0_ = 5% and 7% for the FNN model and *σ*_0_ = 5%, 7% and 9% for the CNN model. For both models, the *s*O_2_ predictions with uncertainty falling within the 7 ± 1% range and higher are slightly conservative, with *P*(*σ*_0_, *η*) higher than the predicted confidence; for the FNN model, the *s*O_2_ predictions with uncertainty *σ*_0_ = 5% are slightly over-confident since *P*(*σ*_0_, *η*) is lower than the confidence (Fig. 5a); while for the CNN model, the results falling within 5 ± 1% are quite close to the diagonal line (Fig. 5b), indicating a well-calibrated DSL model in this regime. In ref. ^13^, the accuracy for *s*O_2_ calculated by LSF was estimated to be within ±4% relative error in a well-controlled in vitro blood calibration experiment, using blood analyser readings as the ground truth. The uncertainty predicted here (~5% *s*O_2_) by DSL agrees reasonably well with the in vitro calibration result.

Figure S4 illustrates the sub-sets of data when *σ*_0_ = 5%, 7% and 9% for both models. Table 1 provides a summary of the fitting parameters, slope, and constant and how many of the total 502 testing data points were counted for by the statistical analysis within each uncertainty range.

**Table 1:** Parameters of the linear fit of ${\it{P}}\left( {{\it{\upsigma }}_0{\mathrm{,}}\;{\it{\upeta }}} \right)$ to theoretical confidence for sO_2_ predictions with different uncertainties (*σ*) by the FNN and CNN models

Models and uncertainties	Slope	Constant	Datasets
The FNN model (*σ*:5% ± 1%)	0.9843	−0.04286	232
The FNN model (*σ*:7% ± 1%)	1.0551	0.01411	230
The CNN model (*σ*:5% ± 1%)	0.9726	0.003070	185
The CNN model (*σ*:7% ± 1%)	0.9955	0.06697	236
The CNN model (*σ*:9% ± 1%)	0.9884	0.09652	75

### *En faces*O_2_ maps with uncertainty quantification

**Fig. 6:**
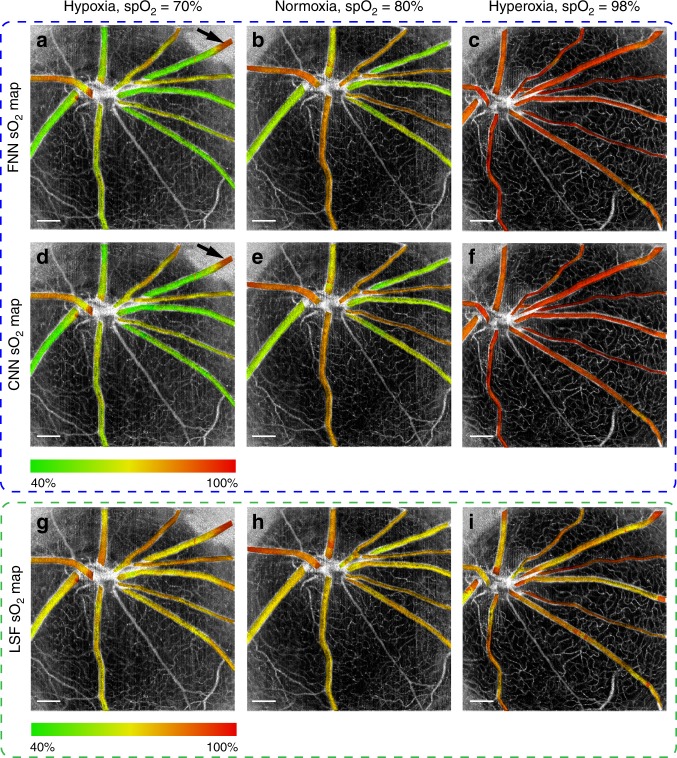
The *en face* *s*O_2_ maps of the testing data for rat retinal oximetry obtained by the FNN **a**–**c**, CNN **d**–**f** and LSF **g**–**i** at hypoxia in **a**, **d** and **g**, normoxia in **b**, **e** and **h**, and hyperoxia in **c**, **f** and **i**. The oximeter *sp*O_2_ readings at hypoxia, normoxia, and hyperoxia are 70%, 80%, and 98%, respectively. Black arrows in **a** and **d** point to a region with inconsistent *s*O_2_ predictions in the same vessel. Scale bar: 500 μm.

**Fig. 7:**
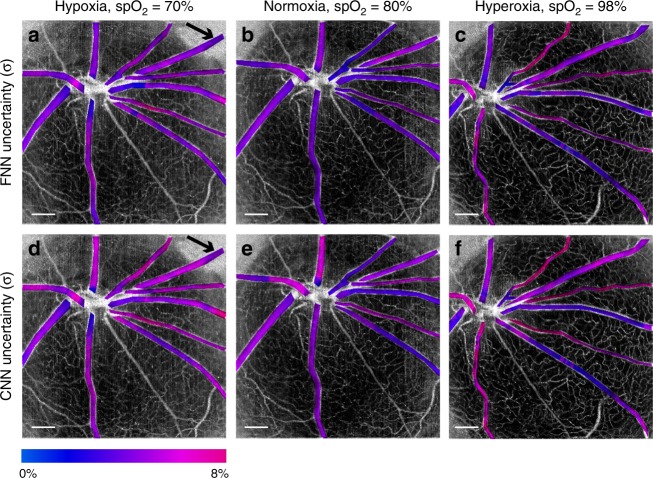
The *en face* uncertainty (*σ*) maps for *s*O_2_ predictions corresponding to Fig. 6a, f. **a**–**c** Uncertainty maps predicted by the FNN model under three ventilation conditions. **d**–**f** Uncertainty maps obtained by the CNN model under three ventilation conditions. Black arrows in **a** and **d** point to a region with inconsistent *s*O_2_ predictions in the same vessel. Scale bar: 500 μm.

After model testing and uncertainty analysis, we used the testing data from ref. ^15^ and applied the FNN and CNN models for retinal-imaging oximetry in comparison to LSF (Fig. 6) under three different ventilation conditions. The oximetry obtained by both the FNN and CNN clearly reflects the *s*O_2_ changes of all vessels from hypoxia, normoxia, to hyperoxia, with the predicted *s*O_2_ of arterioles matching the oximeter *sp*O_2_ readings well. The *s*O_2_ contrast between arteries and veins is also clearly visualized in hypoxia and normoxia. In comparison, the *s*O_2_ predicted by the LSF has less arteriovenous contrast, and the *s*O_2_ changes with increasing ventilation oxygen level are not as significant as those identified by DSL. The estimated arterial *s*O_2_ by LSF at normoxia and hyperoxia are much lower than the ground truth *sp*O_2_ readings. There are also higher variances in the *s*O_2_ results within each individual blood vessel obtained by LSF than by DSL, particularly in Fig. 6g, i. These results clearly indicate the superior robustness and resilience of DSL to variations in experimental conditions and within vessels. Importantly, our DSL models enable direct visualization of the “*en face*” uncertainty maps of the *s*O_2_ predictions in Fig. 7. The FNN and CNN have similar uncertainty estimations on the *s*O_2_ predictions (Fig. 7a–f), consistent with the previous characterization of *σ* at ~5–7% (Fig. 4). Under hypoxia, the *s*O_2_ estimation appears to be inconsistent at the periphery, as indicated by the black arrows in Fig. 6a, d. We attribute this to the extremely poor signal levels at those regions where severe vignetting was present in the raw data (Fig. S5).

### Cross validations of the DSL models

**Fig. 8:**
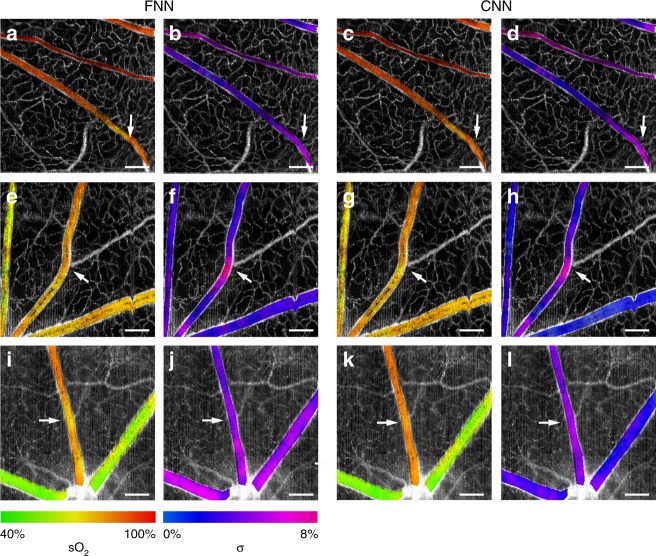
Zoomed-in views of the *s*O_2_ and the corresponding uncertainty maps. **a**–**d** The *s*O_2_ and uncertainty at hyperoxia obtained by the FNN and CNN, respectively, with *sp*O_2_ = 98%. **e**–**h** The *s*O_2_ and uncertainty at normoxia obtained by the FNN and CNN, respectively, with *sp*O_2_ = 76%. **i**–**l** The *s*O_2_ and uncertainty at normoxia obtained by the FNN and CNN, respectively, with *sp*O_2_ = 80%. Scale bar: 500 μm. Arrows point to the locations where *s*O_2_ prediction shows inconsistency within a vessel, which are successfully detected by increased uncertainty levels in the corresponding uncertainty map.

To cross validate our DSL models, we split the training and testing datasets differently and repeated all the analyses for two additional cases. Specifically, Rat 3 in Fig. 3a and Rat 1 in Fig. 3b serve as the testing data in the first case, and Rat 1 in Fig. 3a and Rat 5 in Fig. 3b, in the second case. Together with the original testing case (Fig. 4), the MSEs of all the *s*O_2_ predictions are summarized in Table S2. In all scenarios, the MSEs from DSL are significantly lower than those from LSF by at least 50%. We also repeated the arterial *s*O_2_ testing against the ground truth (Fig. S6–S1) and generated the *s*O_2_ and uncertainty maps (Fig. 8S–11S) in both cross-validation cases. All the DSL *s*O_2_ maps clearly show the arteriovenous contrasts and the rising *s*O_2_ from hypoxia to hyperoxia. All the uncertainty maps also display similar levels of overall confidence.

## Discussion

In this paper, we present a new framework for optical-imaging oximetry based on DSL. The DSL method offers several unique advantages compared to the existing LSF method. First, it bypasses the need for any rigid analytical models and is highly flexible and resilient to experimental variations. We tested DSL on two datasets from two separate vis-OCT experiments and showed that DSL maintained consistent agreement with the ground truth *sp*O_2_ despite the many differences between these two experiments. In contrast, an optimized LSF with the identical parametric settings generated significantly worse accuracy. Second, without the restriction of any rigid models, DSL allows more flexible and efficient use of the data. Here, we demonstrate the effectiveness of using the spectra from both the middle and bottom of the vessels, since both carry *s*O_2_-dependent spectral contrast. Most importantly, DSL not only provides the point estimate of *s*O_2_ but also quantifies the tandem uncertainty of the prediction. Quantifying the statistical uncertainty for each measurement is not possible using the traditional LSF approach; however, it is valuable in assessing the fidelity of each measurement, particularly in clinical applications. We validate the uncertainty quantification by using the reliability diagram, and for the first time to our knowledge, we constructed an uncertainty map of in vivo imaging oximetry showing the estimation confidence by DSL. More generally, our DSL framework presents an attractive data-driven approach for other inverse scattering spectral analyses beyond oximetry.

There are still possible venues to improve the *s*O_2_ and uncertainty map predictions. The overall design of our current approach aims to establish a data-driven regression model directly from spectra to *sp*O_2_ without considering the spatial information present in the raw OCT data. The benefit of this approach is that a conceptually simple 1D neural network can be trained and tested from randomly shuffled spectral OCT data, in which each spectrum is independent. On the other hand, when we reconstruct the *en face* maps, the quality of the maps is also subject to spatially dependent data abnormalities, such as low signal contrast, as shown in Fig. 6 in the periphery region. This is a limitation of our current DSL model that may be addressed by a complex 2D/3D spatio-spectral model that incorporates both oxygen maps as the ground truth^49^ and additional spatial information in our future work. Despite this, our DSL prediction performs well and faithfully follows the physiological rules described in the section on *en face* *s*O_2_ maps. In many cases with sufficient signal contrast, uncertainty prediction does provide reasonable confidence assessments, as shown in a few zoomed-in regions in Fig. 8. The full *en face* maps are provided in Figs. S8–S11. In all these cases, it is shown that the uncertainty levels are higher (i.e., less confidence) in the regions where the *s*O_2_ predictions suffer from inconsistency within the vessel, which justifies the utility of the *en face* uncertainty maps.

The rationale of using pulse oximetry as the arterial *s*O_2_ ground truth is for the ease of clinical translation, since pulse oximetry is a standard-of-care method in clinics. Thus, we expect the same framework to be duplicated in clinical studies. Due to the cardiac cycle, arterial blood has a pulsatile flow pattern, by which pulse oximetry provides consistent and accurate arterial *sp*O_2_, regardless of the measurement sites (e.g., at the ear lobe, fingers, foot (in the case of an infant), or skin with the reflectance mode)^50–52^. Therefore, it is convenient and accurate to use *sp*O_2_ as a ground truth for *s*O_2_ in retinal arteries. For veins, although we do not have the ground truth, our methodology is expected to be valid because the spectroscopic properties of the blood are independent of the arteries or veins but are oxygenation-dependent. The experiments in both datasets changed the oxygen content in the ventilation air, a common approach in physiological studies, to globally modulate the systemic blood oxygenation. By doing so, the network attempts to establish the mapping between the spectral features from the arterial blood and the *sp*O_2_. As long as the venous *s*O_2_ falls within the ranges of the training dataset, the networks are expected to produce reliable prediction since the network is fed with the spectral data without prior knowledge of their arterial or venous origins.

We envision that the method presented here can generate immediate impacts in ophthalmology, as shown in recent preclinical studies on diabetic retinopathy, glaucoma, and retinal vessel occlusion^43–47^. In particular, since the spectroscopic features of haemoglobin are universal in rodents and humans, we expect the framework developed in this work to be rapidly adapted to human vis-OCT data. Beyond the vis-OCT and retina, we believe the DSL-enhanced optical oximetry may find broad applications on other tissue sites, as long as there are sufficient vascular signal contrast and accessible training datasets. More generally, our DSL framework presents an attractive data-driven approach for other inverse scattering spectral analyses beyond oximetry.

## Materials and methods

### Vis-OCT experiments

The vis-OCT systems in refs. ^13^^,15^ had the same spectral range (from 520 to 630 nm), with the same lateral and axial resolutions, estimated to be 15 and 1.7 μm, respectively. The scanning protocol in ref. ^13^ used a raster scan over a 20° square retinal area covering a field of view (FOV) of 2.51 mm × 2.51 mm, with 256 × 256 pixels in each direction at a 25 kHz A-line rate. The exposure time for the spectrometer camera was 37 µs. The entire vis-OCT image stack took 3.3 s to acquire. The scanning protocol in ref. ^15^ was for optical coherence tomography angiography, scanning a 40° square retinal area covering an FOV of 4.37 mm × 4.37 mm, with 400 pixels in the A-line direction and 512 pixels in the B-scan direction at a 50 kHz A-line rate. The exposure time for the spectrometer camera was 17 µs. For the sake of vis-OCT angiography, there were repetitive (5×) unidirectional B-scans of the same cross section, giving a total of 5 × 512 B-scans for each acquisition. The entire vis-OCT image stack took 25.6 s to acquire.

### Spectral extraction and data preprocessing

Wavelength-dependent vis-OCT images were first generated by a short-time Fourier transform (STFT) with 14 equally spaced Gaussian spectral windows in the *k*-space. The wavelength spans from 523.4 to 604.5 nm. The size (FWHM) of the Gaussian window in the *k*-space is 0.32 μm^−1^, corresponding to a bandwidth of ~17 nm at 585 nm. After STFT, a spectrum can be obtained at each 3D vis-OCT voxel. Next, we performed segmentation to isolate the spectra within retinal arterioles^17,18^. Retinal blood vessels are first segmented from the *en face* projection of the 3D vis-OCT image by a threshold-based algorithm^18^; next, all A-lines within the segmented retinal arterioles are shifted in the axial direction in reference to the retinal surface and randomly shuffled. Here, we applied the rolling average, which analyses data by creating a series of averages of different subsets, on the shuffled A-lines before extracting the input spectra for the DSL models. Specifically, a rolling average of 100 shuffled A-lines in ref. ^13^ and 250 shuffled A-lines in ref. ^15^ was performed with 50 and 125 rolling step sizes, respectively. Because the major vessels are located on top of the retina, the signal within the vessels can be averaged in reference to the retinal surface to generate one spectrum. We located the bottom vessel wall^13,25^ and averaged signals within ±16.6 μm to generate the vessel bottom spectrum. We then averaged the signal from ~25 ± 8.31 and 41.6 ± 8.31 μm above the bottom vessel wall to generate the vessel centre and top spectra, respectively. For DSL, the two spectra from the vessel bottom and centre were concatenated as a single spectrum signal. Finally, each individual spectrum input was normalized by the mean of the combined signal from all three spectra (vessel bottom, center, and top) to ensure similar scaling of all datasets before neural network training.

### Principle of least-squares fitting

Vis-OCT uses ballistic photon and coherence gating to localize the optical signal within a tissue volume. At the bottom of the vessel wall, light double-passing through the vessel lumen gives rise to the detectable spectral contrast, which can be analytically formulated based on Beer’s law^13^

2 $${\mathop{I}\nolimits} \left( {s{\mathop{\rm{O}}\nolimits} _2|\lambda ,z} \right) = {\mathop{I}\nolimits} _0\left( \lambda \right)\sqrt {R_0r\left( \lambda \right)} {\mathrm {e}}^{ - \left[ {{\mathop{\rm{sO}}\nolimits} _2 \times \mu _{{\mathrm {HbO}}_2}\left( \lambda \right) + \left( {1 - {\mathop{\rm{sO}}\nolimits} _2} \right) \times \mu _{{\mathrm {Hb}}}\left( \lambda \right)} \right]z}$$

where I_0_(*λ*) is the spectrum of the light source; *R*_0_ is the reflectance of the reference arm and assumed to be a constant; and *r*(*λ*) (dimensionless) is the reflectance at the vessel wall, the scattering spectrum of which can be modelled by a power law under the first Born approximation *r*(*λ*) = *Aλ*^*−α*^, with *A* being a dimensionless constant and *α* modelling the decaying scattering spectrum from the vessel wall. The optical attenuation coefficient *μ* (mm^−1^) combines the absorption (*μ*_a_) and scattering coefficients (*μ*_s_) of the whole blood, which are both wavelength- and sO_2_-dependent:

3 $$\mu = \mu _{\mathrm {a}} + W\mu _{\mathrm {s}}$$

where *W* is a scaling factor for the scattering coefficient in a range from 0 to 1^6,17,53^. The subscripts Hb and HbO_2_ denote the contribution from the deoxygenated and oxygenated blood, respectively. *z* denotes the light-penetration depth.

To estimate *s*O_2_, the traditional approach applies the least-squares procedure that fits the vis-OCT spectral measurement to the analytical model by minimizing the total squares of the error, as illustrated in Fig. 1a:

4 $$\begin{array}{*{20}{c}} {} \qquad{\qquad\qquad\qquad ^{\min }_{s{\mathop{\rm{O}}\nolimits} _2,A,\alpha }} \end{array}\mathop {\sum}\limits_\lambda {\left\| {\log \left[ {{\mathop{I}\nolimits} _{\mathrm {m}}\left( \lambda \right)} \right] - {\mathrm {log}}\left[ {I\left( {s{\mathop{\rm{O}}\nolimits} _2,A,\alpha |\lambda ,z = D} \right)} \right]} \right\|} ^2$$

where *I*_m_ is typically taken as the vis-OCT spectral measurements extracted from the bottom of the vessel to maximize the spectroscopic contrast. The LSF model parameters can be optimized by adjusting the scaling factor *W* and the spectral wavelength range. We ensured that the whole spectral range covers ~550–585 nm to include the most dominant spectral contrast between oxygenated and deoxygenated blood. The optimization minimized the MSE between the predicted *s*O_2_ and the ground truth *sp*O_2_. Figure S12 shows the MSE with varying *W* and fitting spectral range for the combined data from refs. ^13^,^15^ and for data from each reference separately. Since the DSL models are trained by a mixture of both data sources, to ensure a fair comparison, we used the optimized LSF model for the combined datasets, where *W* = 0.12 and the fitting spectral range is 548–586 nm, respectively (Fig. S12a).

In this analytical model, two free parameters (*A*, *α*), in addition to the unknown *s*O_2_ level, are introduced to more accurately capture realistic biophysical interactions. However, in practice, these two parameters cannot fully capture all the experimental variabilities. While other models may reduce the free parameters to avoid overfitting^14,15,19,20,24,54^, this approach in general is nonetheless rigid and over-simplified with respect to the actual experiments.

### Principle of deep spectral learning

In DSL, instead of using a rigid analytical model, we train a neural network to link the spectral measurements and the independently measured *s*O_2_ labels, as illustrated in Fig. 1b. By doing so, DSL bypasses the need for the parametric tuning and model simplification and approximation needed in fitting the analytical models. Furthermore, by removing the restrictions imposed by the analytical model, DSL allows utilizing multiple sets of spectral measurements taken at different depths and enables a more holistic spectral-*s*O_2_ analysis. Specifically, we demonstrate high-quality predictions using concatenated spectra data from both the bottom and the center of the vessels in vis-OCT. Because the pulse oximeter measures the systemic arterial *s*O_2_, the same as with the retinal arterioles, we use the retinal arterial spectra as the training input paired with the independently measured pulse oximeter *s*O_2_ (*sp*O_2_) as the ground truth label. After training, the network makes predictions for both arterials and veins.

In addition to *s*O_2_ prediction, a major important feature of our data-driven DSL method is to quantify the uncertainty for *each* prediction. To do so, we specifically design the loss function for training the network to properly capture the underlying statistics of the data. The commonly used loss function, that is, the mean squared error (MSE), assumes a homogeneous Gaussian distribution of the errors in the predictions. This assumption severely limits its ability to adapt different types of spectral data variations (e.g., spectral signal outliers, non-uniform noise, and unevenly sampled data) that are inevitably *inhomogeneous*. To account for this, we design a customized loss function derived from a *heterogeneous* Gaussian distribution model. Using the training data set (*I*_*i*_, [*sp*O_2_]_*i*_), *i* = 1, 2, …, *N*, where *I*_*i*_ and [*sp*O_2_]_*i*_ are the *i*th vis-OCT spectral measurement and the ground truth pulse oximeter *sp*O_2_, respectively, our loss function *L*_G_(*w*) is

5 $${L}_{{\mathrm {G}}}\left( w \right) = \mathop {\sum}\limits_{i = 1}^N {\left\{ {\frac{{\left( {\left[ {s{\mathrm {O}}_2} \right]_i\left( w \right) - \left[ sp{{\mathrm {O}}_2} \right]_i} \right)^2}}{{\sigma _i^2\left( w \right)}} + \log \left[ {\sigma _i^2\left( w \right)} \right]} \right\}}$$

where [*s*O_2_]_*i*_ and *σ*_*i*_ denote the neural network predicted mean and standard deviation, respectively, of the underlying Gaussian distribution for the *i*^th^ training data pair; *w* is the learned neural network weights. The main idea of this loss function assumes that the prediction made on each spectrum follows a distinct Gaussian distribution, and the network is trained to predict the underlying mean and standard deviation^55^. Accordingly, the standard deviation *σ* quantifies the uncertainty for each *s*O_2_ prediction.

We investigate two neural network models, including an FNN and a CNN model, the network architectures of which are shown in Fig. 2a, b, respectively. The detailed descriptions of the network architectures are included in the following section.

### Network architectures and training

The first FNN model, as illustrated in Fig. 2a, concatenates two spectra from both the bottom and centre of the vessels with a size of 1 × 28 as the input. The output predicts both the mean *s*O_2_ level and the uncertainty (measured by the standard deviation) in two output channels, both of which are single values in units of volume percentage of the blood being oxygenated. The model has two hidden layers, each having 24 units. We use the ReLU-activation function in the two inner layers and the sigmoid activation function in the final layer to normalize the predictions between 0% and 100%.

The second CNN model, as illustrated in Fig. 2b, takes the same input as the FNN model and predicts the *s*O_2_ with uncertainty. The model has two convolutional layers, with each having a filter size of 3 and a filter number of 30, and one flatten layer. We use the ReLU-activation function in the two convolutional layers and the sigmoid activation function in the final layer.

All data processing and network training are implemented in Python using the TensorFlow/Keras library. Both models were trained with an initial learning rate of 2.5 × 10^−3^, and we gradually decreased the rate by 1/(1 + *αN*), where *N* is the epoch number and *α* is a decay rate set to 0.01. The same total epoch number of 2000 with the same batch size of 50 ensured that the learning curve could reach a plateau. We set the validation split ratio as 0.2 and selected the model with the minimum validation loss as the optimal one for following *s*O_2_ prediction.

### Loss function for uncertainty quantification

In the proposed DSL model, denoted by its network weights *w*, the network makes estimation on both the mean and the standard deviation of *s*O_2_ given the input spectral measurement. Assuming that the *s*O_2_ of all retinal arterioles of a rat at each particular oxygenation status satisfy different Gaussian probability distributions,

6 $${\it{p}}_{\it{w}}\left( {\left. {{\mathop{\rm{sO}}\nolimits} _2} \right|I_i} \right) = {\it{N}}\left( {{\mathop{\rm{sO}}\nolimits} _2,\sigma ^2} \right)$$

where the mean and the standard deviation of the Gaussian distribution are denoted as *s*O_2_ and *σ*, respectively, and *N* denotes the normal distribution. The neural network learns a highly complex nonlinear function. During the training, the network weights, *w*, are estimated by maximizing the joint likelihood over *N* training data pairs:

7 $$w = {\mathrm{arg}}\begin{array}{*{20}{c}} {} \\ {\max } \\ {w\,\!\! \in F} \end{array}{\mathrm{likelihood}}\left( w \right) = {\mathrm{arg}}\begin{array}{*{20}{c}} {} \\ {\max } \\ {w\,\!\! \in F} \end{array}\mathop {\prod}\limits_{i = 1}^N {\frac{1}{{\sqrt {2\pi \sigma _i^2\left( w \right)} }}{\mathrm {e}}^{ - \frac{{\left( {[s{\mathrm {O}}_2]_i\left( w \right) - \left[ {sp{\mathrm {O}}_2} \right]_i} \right)^2}}{{2\sigma _i^2\left( w \right)}}}}$$

Equivalently, the customized Gaussian loss function *L*_G_ (*w*) is minimized when training the DSL models in Eq. (5).

### Reconstruction of *en face* maps of sO_2_ and uncertainty (σ)

To reconstruct a 2D *en face* map for *s*O_2_ or uncertainty (*σ*), we applied the same spatial signal averaging procedure as described in “Spectral extraction and data preprocessing” section, but this averaging was done pixel-wisely within each blood vessel. We first segmented the *en face* vessel area manually. For each pixel from the 2D *en face* map within the segmented blood vessel, an arteriole or a venule, its depth-dependent spectra were generated by averaging spectral signals from its 100 (in the first literature) or 250 (in the second literature) nearest neighbours based on the Euclidian distance. Then, these spectra would be input into the FNN or CNN model to predict a *s*O_2_ with uncertainty for this pixel. Next, the above two steps would be iterated pixel-wisely until all pixels within this particular blood vessel had predicted *s*O_2_ and uncertainty. Finally, the above three steps would be iterated until all arterioles and venules of the rat retina had predicted *s*O_2_ and uncertainty for displaying their 2D *en face* maps. To generate Figs. 6 and 7, we applied an algorithm based on the HSV (hue, saturation, and value) colour model, where the predicted *s*O_2_ or uncertainty corresponds to the image hue, and the angiography signal intensity corresponds to the image value and saturation.

## Supplementary information

Supplementary Materials
