# Supplementary material for: Deep spectral learning for label-free optical imaging oximetry with uncertainty quantification
Source: Light Sci Appl. 2019 Nov 20;8:102. doi: 10.1038/s41377-019-0216-0 (PMC6864044; doi:10.1038/s41377-019-0216-0)
Supplement: Supplementary file 1 — Supplementary Materials [file 41377_2019_216_MOESM1_ESM.docx]

Supplemental Information

Deep spectral learning for label-free optical imaging oximetry with uncertainty quantification

Rongrong Liu,^1^ Shiyi Cheng,^2^ Lei Tian,^2^ Ji Yi,^2,3,4,*^

^1^Department of Biomedical Engineering, Northwestern University, Evanston, IL, 60208, USA

^2^Department of Electrical and Computer Engineering, Boston University, Boston, MA 02215, USA

^3^Department of Biomedical Engineering, Boston University, Boston MA, 02215, USA

^4^Department of Medicine, Boston University School of Medicine, Boston Medical Center, Boston MA, 02118, USA

*Corresponding author: [jiyi@bu.edu](mailto:jiyi@bu.edu)

**Table of content**

| Item | Content | Page |
| --- | --- | --- |
| DSL model generalization | | |
| 1 | Table S1. MSEs by FNN, CNN, and LSF on different generalization cases. | 2 |
| 2 | Fig. S1. Training and testing only on data from Ref. 13 | 2 |
| 3 | Fig. S2. Training and testing only on data from Ref. 15 | 3 |
| 4 | Fig. S3. Training on Ref. 13, testing on Ref. 15 | 3 |
| Validation of prediction uncertainty | | |
| 5 | Fig. S4. Illustration of the uncertainty validation | 4 |
| Image signal analysis | | |
| 6 | Fig. S5. Example of images and spectra with low signal level | 4 |
| Cross validation analysis | | |
| 7 | Table S2. MSEs for all the cross validation test cases | 5 |
| 8 | Fig. S6. Arterial sO_2_ prediction in cross validation case 1 | 5 |
| 9 | Fig. S7. Arterial sO_2_ prediction in cross validation case 2 | 6 |
| 10 | Fig. S8. Predicted sO_2_ map by cross validation case 1 | 7 |
| 11 | Fig. S9. Predicted uncertainty map by cross validation case 1 | 7 |
| 12 | Fig. S10. Predicted sO_2_ map by cross validation case 2 | 8 |
| 13 | Fig. S11. Predicted uncertainty map by cross validation case 2 | 8 |
| LSF model optimization | | |
| 14 | Fig. S12. Illustration of the LSF model optimization | 9 |

Table S1: Summary of MSEs for FNN, CNN, and LSF on different generalization cases.

|  | Training data | Testing data | FNN | CNN | LSF |
| --- | --- | --- | --- | --- | --- |
| Case 1 | Ref. 13, Ref. 15 | Ref. 13, Ref. 15 | 0.003539 | 0.003200 | 0.01358 |
| Case 2 | Ref. 13 | Ref. 13 | 0.004866 | 0.004749 | 0.01626 |
|  | Ref. 15 | Ref. 15 | 0.002101 | 0.001895 | 0.007128 |
| Case 3 | Ref. 13 | Ref. 15 | 0.02175 | 0.04954 | 0.2189 |

Case 2, Training and testing on data from Ref. 13


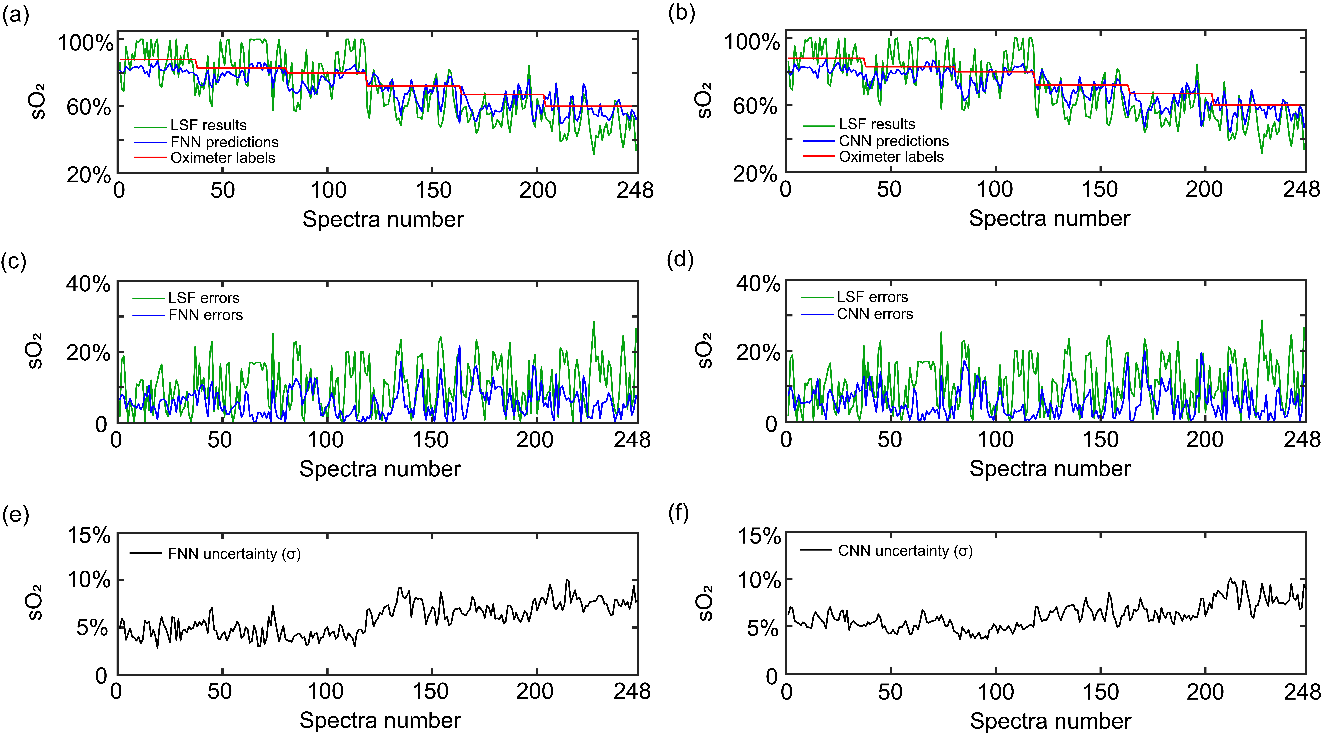


Fig. S1. Rat retinal arteriolar sO_2_ of the testing data at different ventilation conditions predicted by the FNN, CNN, and LSF models. Predicted sO_2_ by FNN (a) and CNN (b), compared with the oximeter spO_2_ readings and LSF calculations. Errors of predicted sO_2_ by the FNN (c) and the CNN model (d) as compared to the LSF results. Predicted uncertainties of sO_2_ by the FNN (e) and the CNN model (f) measured by standard deviations (σ). The DSL training and LSF optimization was conducted on the data from Ref. 13 only, and the testing data are from Ref. 13 as well.

Case 2, Training and testing on data from Ref. 15


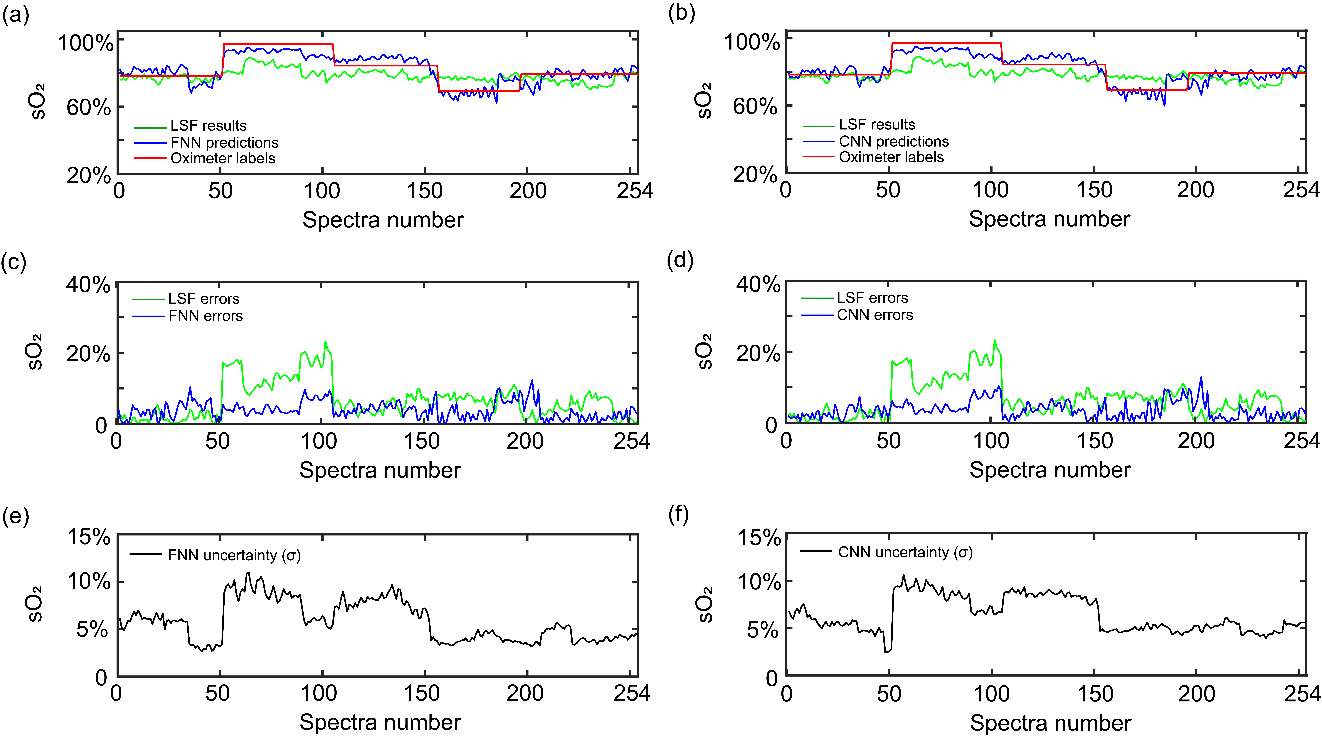


Fig. S2. Rat retinal arteriolar sO_2_ of the testing data at different ventilation conditions predicted by the FNN, CNN, and LSF models. Predicted sO_2_ by FNN (a) and CNN (b), compared with the oximeter spO_2_ readings and LSF calculations. Errors of predicted sO_2_ by the FNN (c) and the CNN model (d) as compared to the LSF results. Predicted uncertainties of sO_2_ by the FNN (e) and the CNN model (f) measured by standard deviations (σ). The DSL training and LSF optimization was conducted on the data from Ref. 15 only, and the testing data are from Ref. 15 as well.

Case 3, Training on data from Ref. 13, testing on Ref. 15


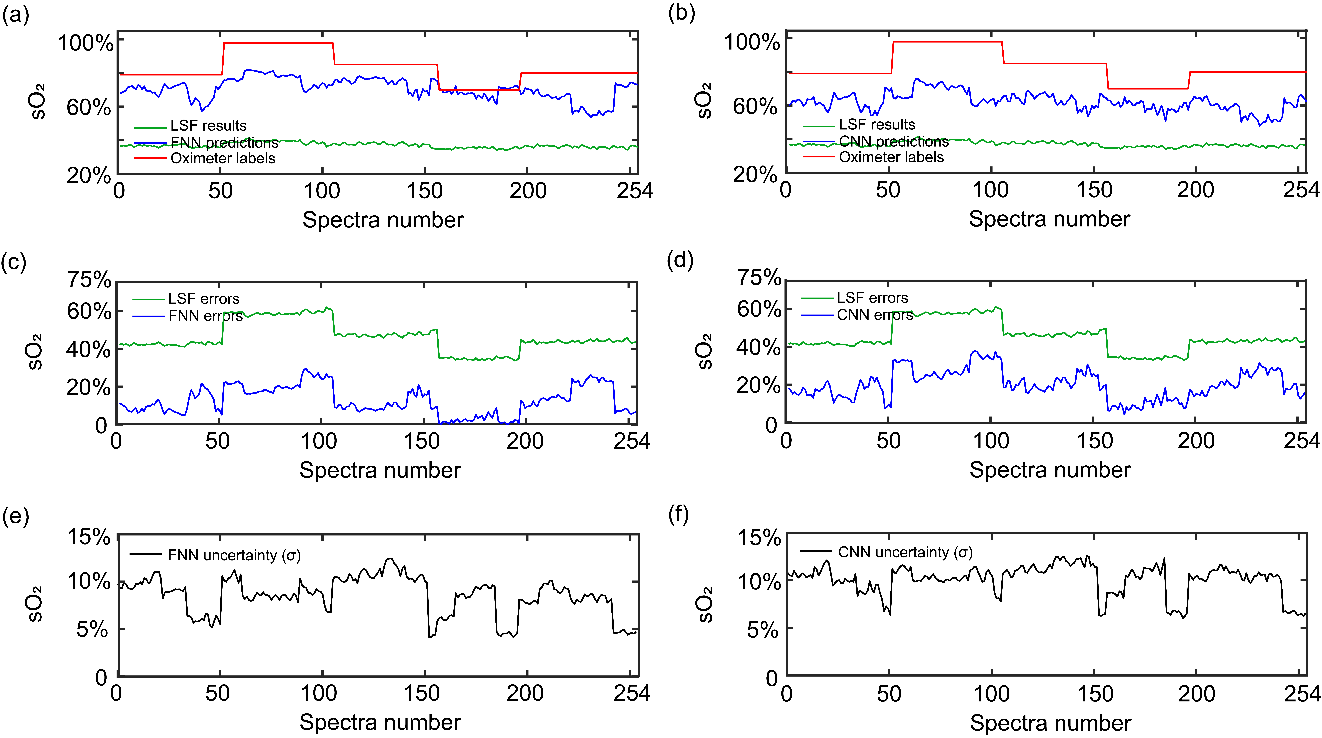


Fig. S3. Rat retinal arteriolar sO_2_ of the testing data at different ventilation conditions predicted by the FNN, CNN, and LSF models. Predicted sO_2_ by FNN (a) and CNN (b), compared with the oximeter spO_2_ readings and LSF calculations. Errors of predicted sO_2_ by the FNN (c) and the CNN model (d) as compared to the LSF results. Predicted uncertainties of sO_2_ by the FNN (e) and the CNN model (f) measured by standard deviations (σ). The DSL training and LSF optimization was conducted on the data from Ref. 13, but the testing data are from Ref. 15.


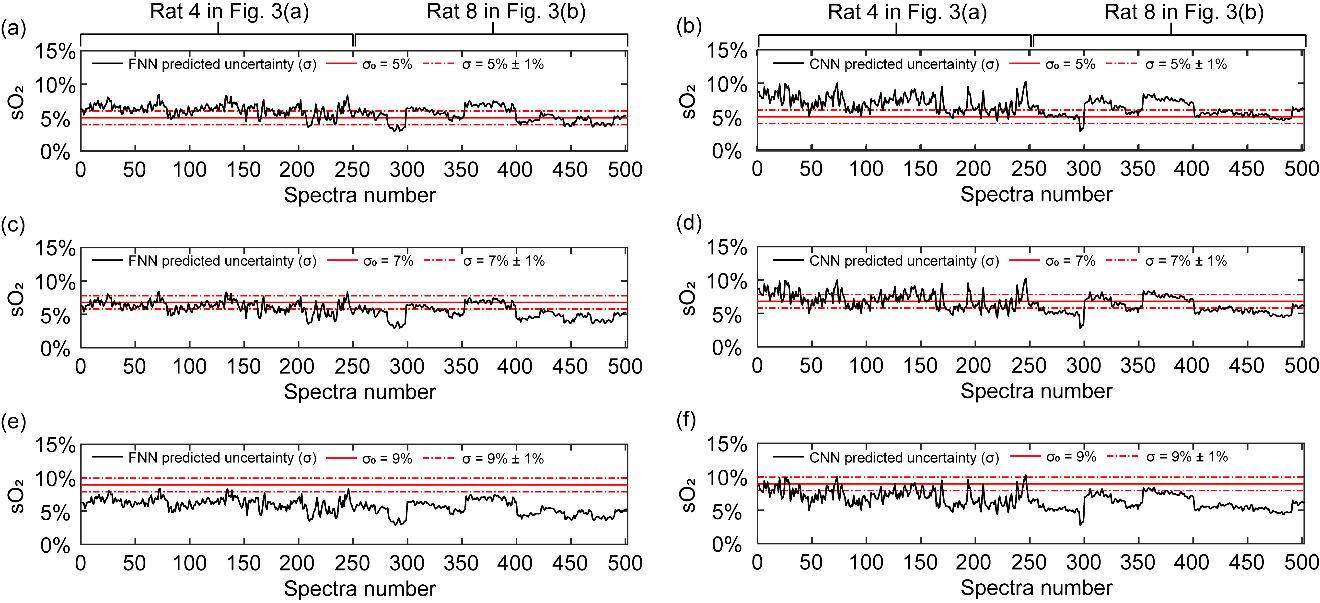


Fig. S4: Illustrations of the sub-set of data selected when σ_0_ = 5%, 7% and 9% in the FNN model: (a), (c), and (e); and the CNN model: (b), (d), and (f).


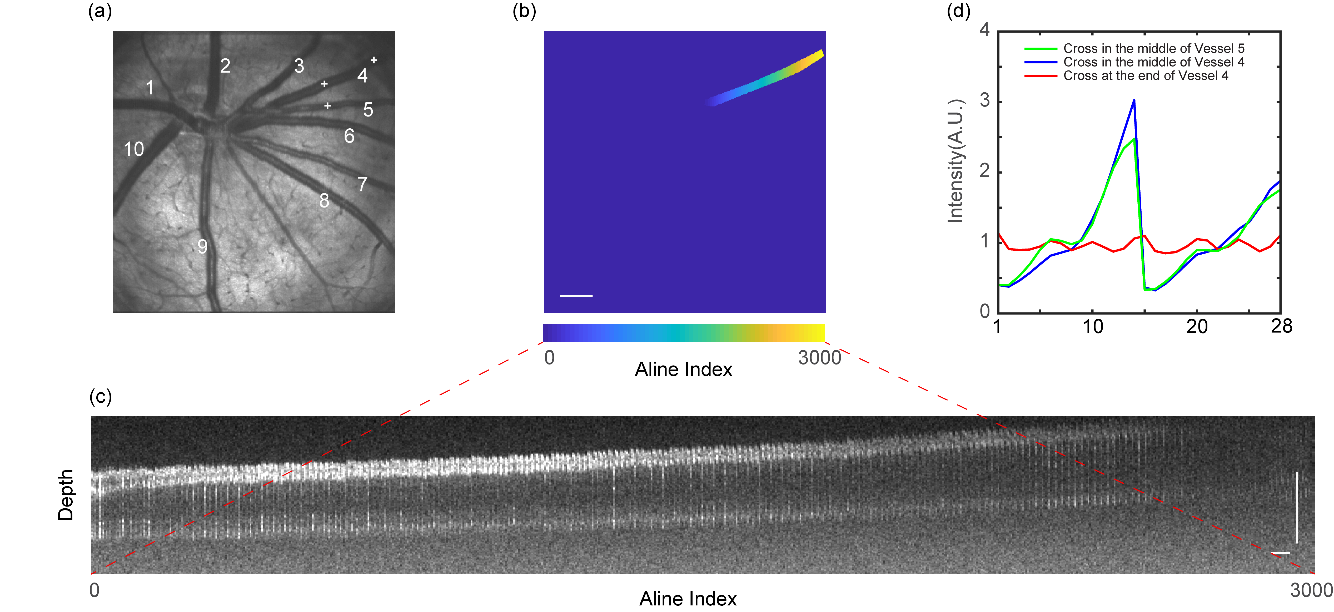


Fig. S5. (a) OCT structural *en face* map of Fig. 6(a) in the main text. (b) Index map of A-lines in Vessel 4 from fovea center to vessel periphery. (c) A-lines in Vessel 4 sorted according to the index in (b). (d) Input spectra (features) from locations of Vessel 4 and Vessel 5 marked by the blue crosses in panel (a). Scale bar: 500 μm.

Table S2: Summary of the MSE for all cross validation tests

|  | Training data (in Fig. 3) | Testing data (in Fig. 3) | FNN | CNN | LSF |
| --- | --- | --- | --- | --- | --- |
| Test 1 (presented in the manuscript) | Rat 1,2,3 in Ref. 13  Rat 1-7 in Ref. 15 | Rat 4 in Ref. 13  Rat 8 in Ref. 15 | 0.003539 | 0.003200 | 0.01358 |
| Test 2 | Rat 1, 2, 4 in Ref. 13  Rat 2-8 in Ref. 15 | Rat 3 in Ref. 13  Rat 1 in Ref. 15 | 0.004105 | 0.004363 | 0.01017 |
| Test 3 | Rat 2-4 in Ref. 13  Rat 1-4, 6-8 in Ref. 15 | Rat 1 in Ref. 13  Rat 5 in Ref. 15 | 0.004864 | 0.005006 | 0.01554 |


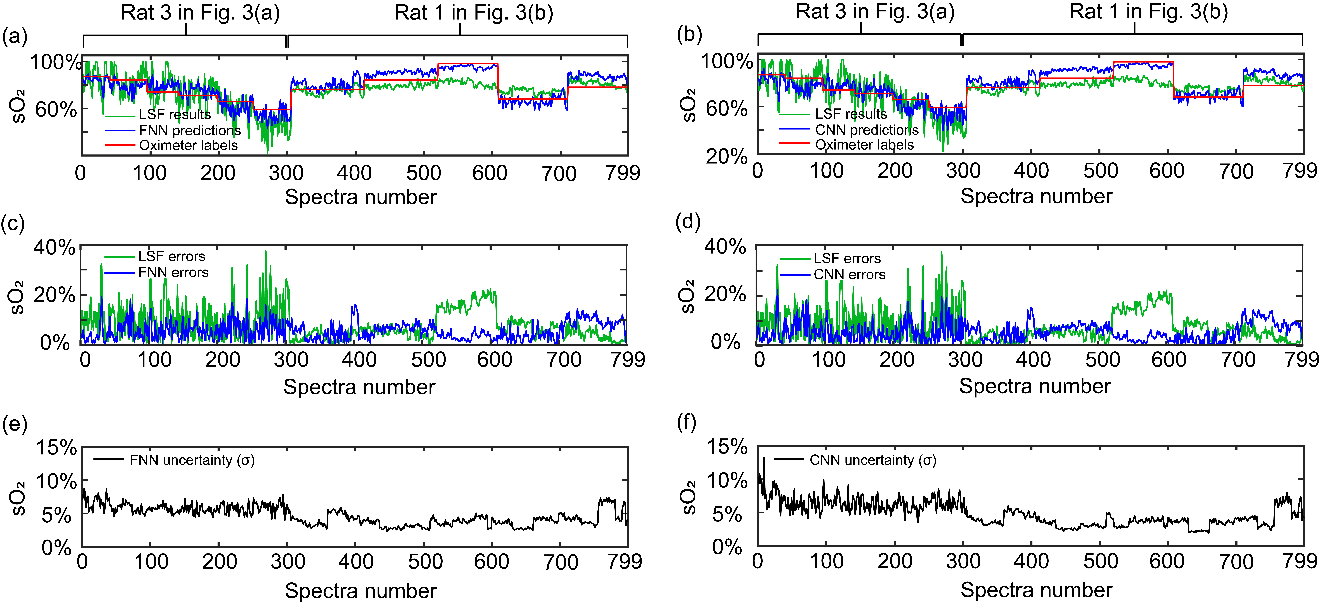


Fig. S6. Rat retinal arteriolar sO_2_ of the testing data at different ventilation conditions predicted by the FNN, CNN, and LSF models. Predicted sO_2_ by FNN (a) and CNN (b), compared with the oximeter spO_2_ readings and LSF calculations. Errors of predicted sO_2_ by the FNN (c) and the CNN model (d) as compared to the LSF results. Predicted uncertainties of sO_2_ by the FNN (e) and the CNN model (f) measured by standard deviations (σ). The first 310 spectra were from Ref. 13, and the rest were from Ref. 15.


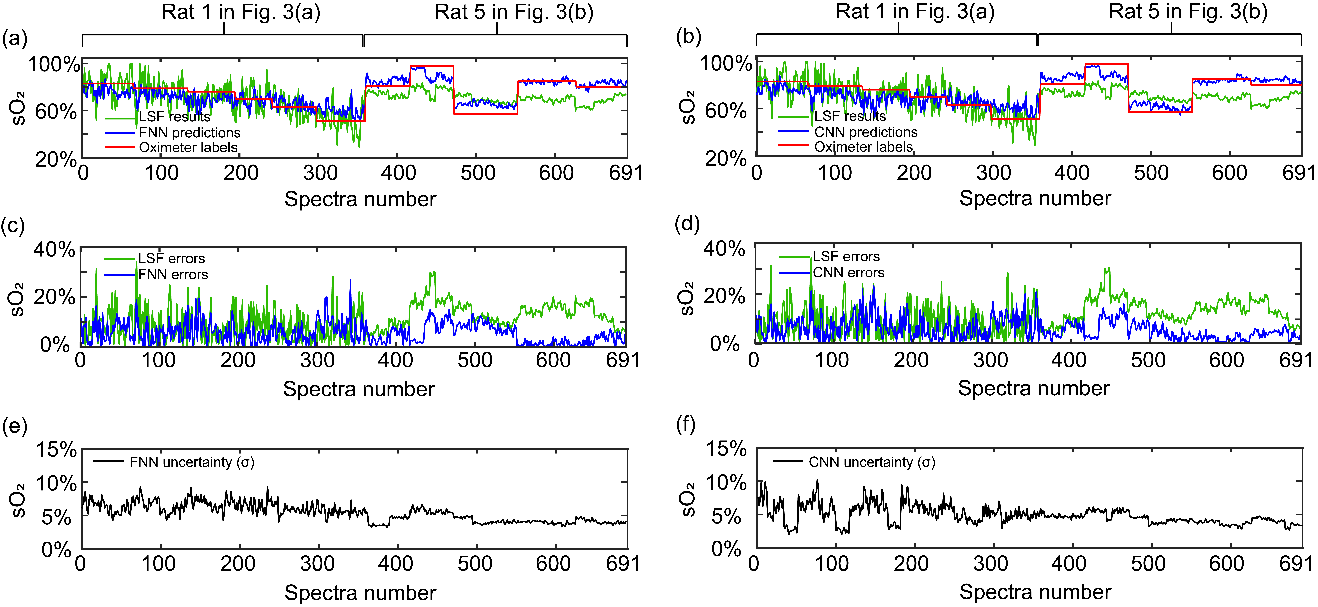


Fig. S7. Rat retinal arteriolar sO_2_ of the testing data at different ventilation conditions predicted by the FNN, CNN, and LSF models. Predicted sO_2_ by FNN (a) and CNN (b), compared with the oximeter spO_2_ readings and LSF calculations. Errors of predicted sO_2_ by the FNN (c) and the CNN model (d) as compared to the LSF results. Predicted uncertainties of sO_2_ by the FNN (e) and the CNN model (f) measured by standard deviations (σ). The first 350 spectra were from Ref. 13, and the rest were from Ref. 15.


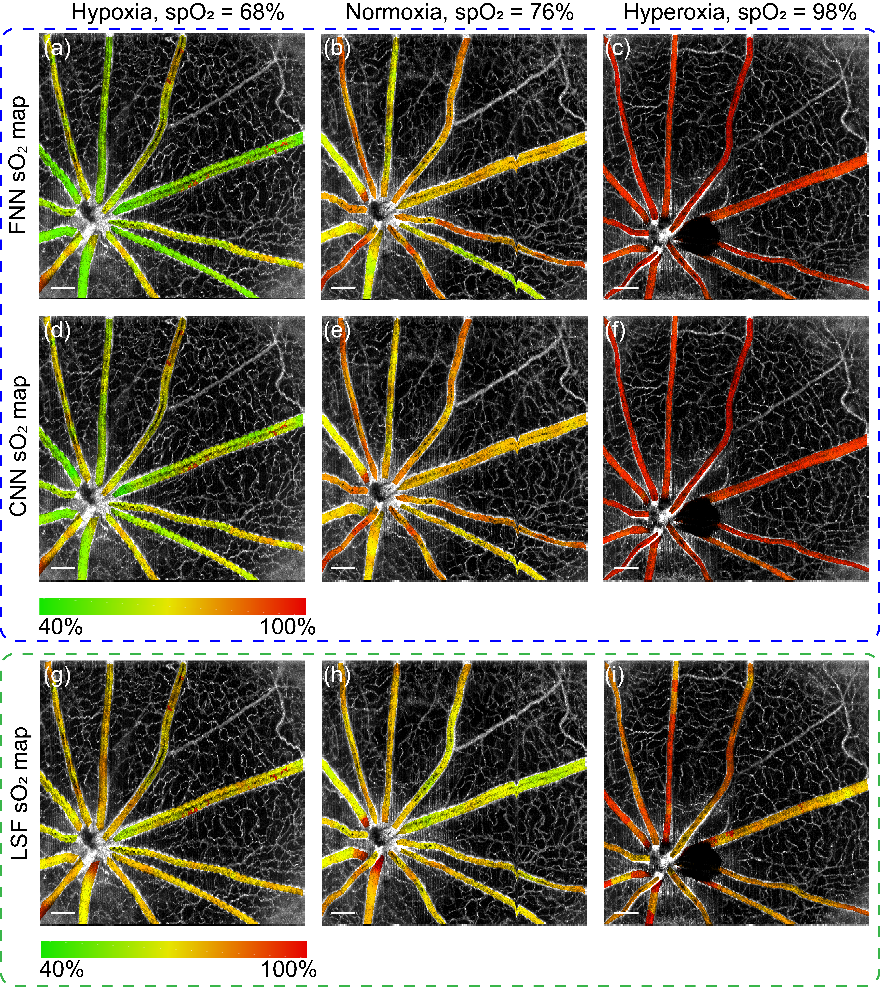


Fig. S8. The *en face* sO_2_ maps of the testing data for rat retinal oximetry by FNN (a-c), CNN(d-f) and LSF(g-i), at hypoxia in (a), (d) and (g), normoxia in (b), (e) and (h), and hyperoxia in (c), (f) and (i). Scale bar: 500 μm. The data is from Rat 1 in Fig. 3(a).


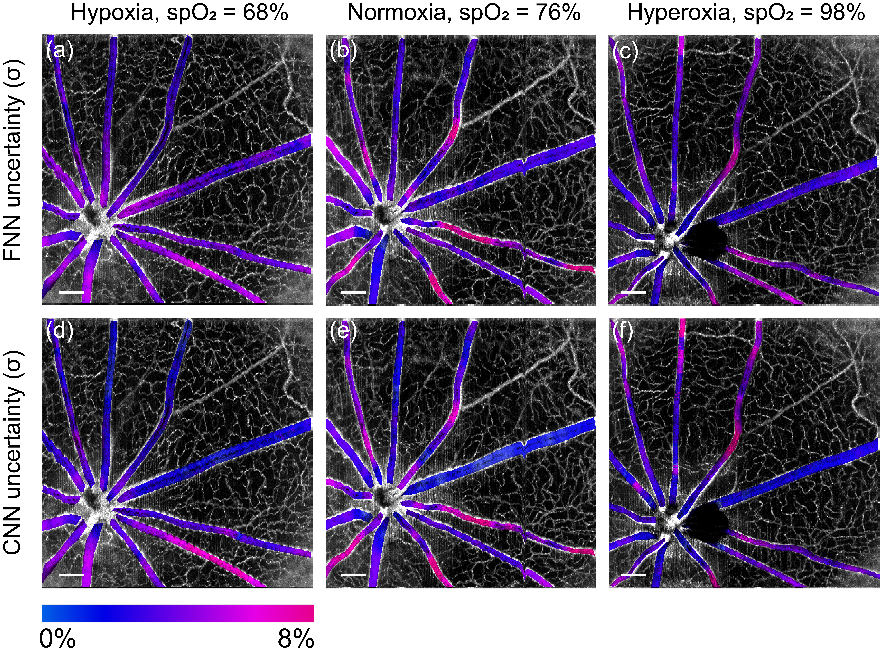


Fig. S9. The *en face* uncertainty (σ) maps for sO_2_ predictions corresponding to panel (a-f) in supplementary Fig. 9. (a-c) Uncertainty maps predicted by the FNN model at three ventilation conditions. (d-f) Uncertainty maps by the CNN model at three ventilation conditions. Scale bar: 500 μm.


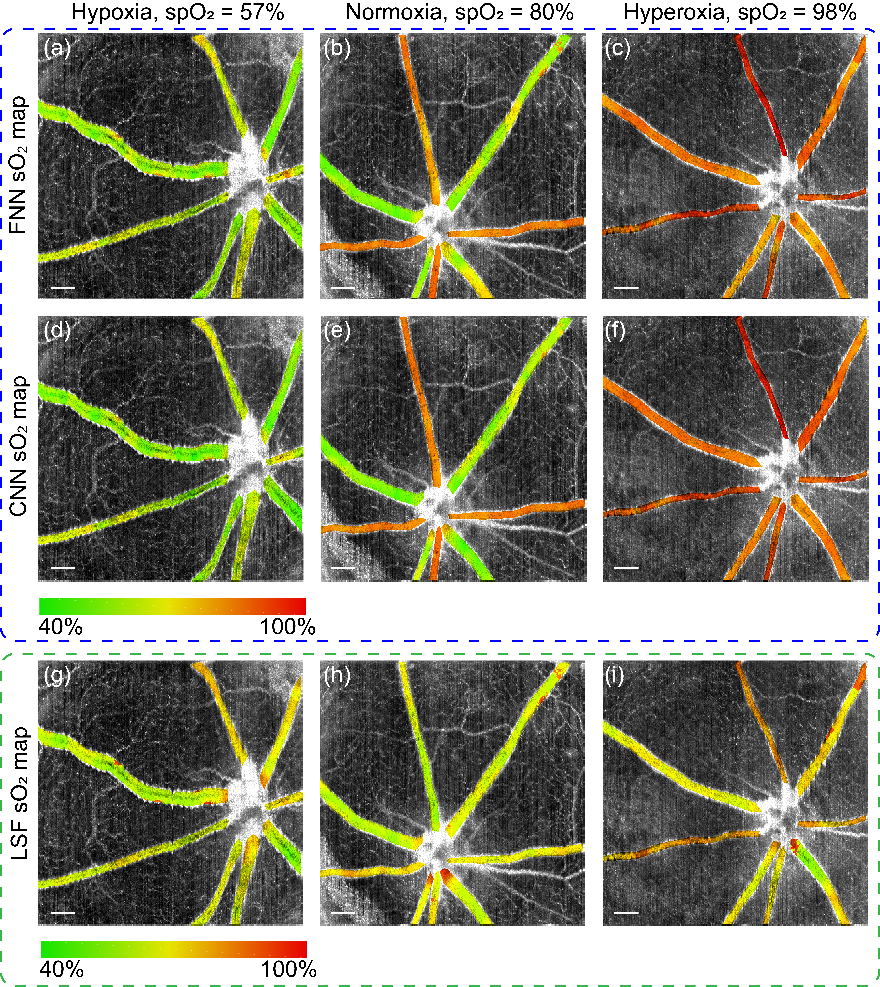


Fig. S10. The *en face* sO_2_ maps of the testing data for rat retinal oximetry by FNN (a-c), CNN(d-f) and LSF(g-i), at hypoxia in (a), (d) and (g), normoxia in (b), (e) and (h), and hyperoxia in (c), (f) and (i). Scale bar: 500 μm. The data is from Rat 5 in Fig. 3(b).


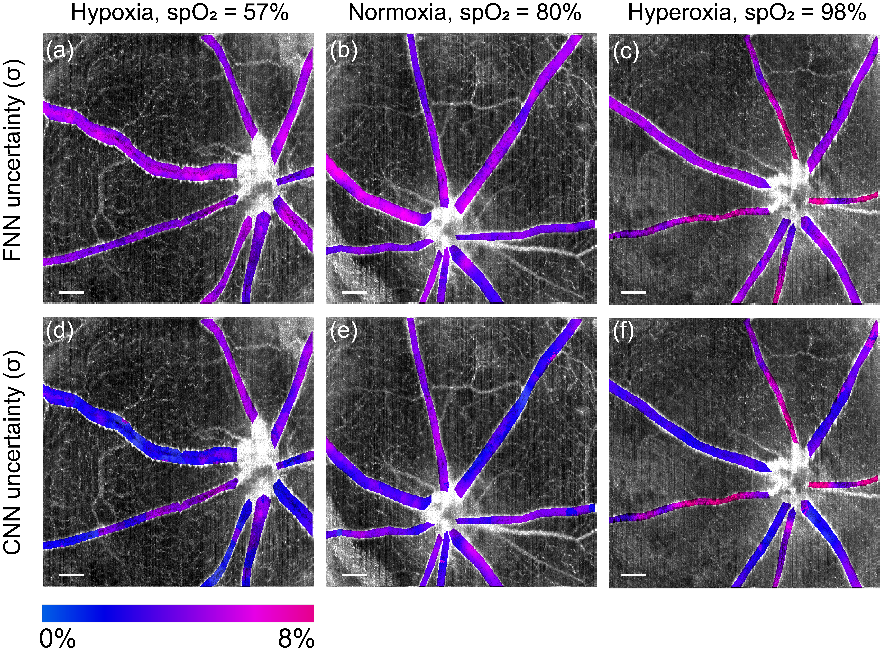


Fig. S11. The *en face* uncertainty (σ) maps for sO_2_ predictions corresponding to panel (a-f) in Supplemental Fig. 10. (a-c) Uncertainty maps predicted by the FNN model at three ventilation conditions. (d-f) Uncertainty maps by the CNN model at three ventilation conditions. Scale bar: 500 μm.


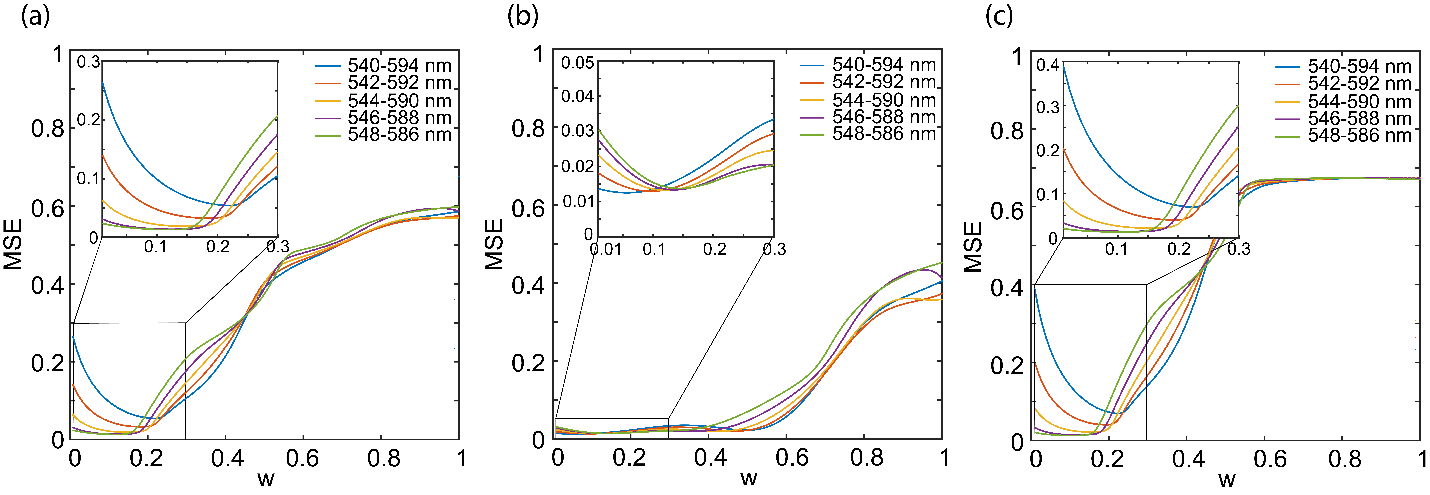


Fig. S12: Illustrations of how the scaling factor *W* and the spectral range to do LSF affect the MSE of calculated sO_2_ using all data from both Ref. 13 and Ref. 15 in (a), using all data only from Ref. 13 in (b), and using all data only from Ref. 15 in (c). On top left of each figure are blow-up boxes of the regions cropped on the bottom left.
